# Suggestions Relative to the Treatment of Rabies Contagiosa

**Published:** 1815-04

**Authors:** William Guy

**Affiliations:** Chichester


*For the Medical and Physical Journal.*

### *Suggestions relative to the Treatment of Rabies Contagiosa;* by Mr. W. Guy.

"Non decet equidem priorum vestigia contemnere; sed permissum et nobis est et invenire aliquid, et mutare, et relinquere. Qui ante nos scripserunt, non domini sed duces nostri fuerunt; crescunt hæc indies, et inventuris inventa non obstant: multum subinde etiam futuris relinquitur."

IT has been well remarked, that "if the prospect of that which remains to be done, after the labours of so many ages, tends to abate hope, and to discourage endeavour; yet it should be remembered, that no man can determine the measure of success which may be connected with industrious research and zealous exertion; and that whatever may be the degree of advancement of which the medical art is capable, he who endeavours to perfect it, has the satisfaction at least of knowing, that he is fulfilling his duty, when his efforts are directed to alleviate the sorrows, and diminish the sufferings, of mankind.

By such feelings the physician is undoubtedly greatly supported, when, in his professional capacity, he is encountering the numerous and dreadful calamities to which human nature is subject; and in no case can he stand in greater need of such consolation, than when contending with the malady which forms the subject of this paper. In truth, a more distressing situation can hardly offer itself to a feeling mind, than that in which a physician is placed, when called upon to attend a patient labouring under Rabies Contagiosa: for, whilst, on the one hand, he feels himself not justified in pursuing plans which have uniformly failed; yet, on the other, he is full of despair, not knowing of any more likely to succeed. Hence, vacillating between opposite plans, and contending opinions, he hesitates, whether, with Dr. Rush, he he should, in order to save his patient's life, perform tracheotomy; or whether, with Dr. Shoolbred, he should bleed him nearly to death, for the same purpose. Whether immerse him in a hot bath of oil or water, or nearly drown him in a cold one. Whether he should blister, galvanize, or electrify; or, by the aid of opium, drown his senses, and lay him to sleep for ever. And probably, after having used his best endeavours for his patient's recovery, his practice may be criticised by some, because he, perhaps, omitted to call into his aid mercury or antimony, copper or zinc, iron or arsenic, bark or camphor, musk or assafœtida, &c. &c. each of which has had an advocate. Under such perplexities, it is surely natural to endeavour to think for ourselves, and, if possible, to emancipate ourselves from such shackles; and the more so when we take it into our consideration, that not one of the many remedies hitherto suggested have ever saved a single patient.

Strongly impressed, as I confess myself to be, with these considerations, I hope my motives in this address will not be misconstrued; and, as I renounce all intention of dictating what steps ought to be pursued in the treatment of this hitherto-fatal malady, so I trust, by submitting for further consideration the plan of treatment I should certainly adopt, if called in to the case of a rabid patient, I shall, in the eyes of the candid at least, stand acquitted of voluntary presumption, and that they will the rather attribute it to its true source—the discharge of a professional duty; but, before I proceed to this step, I shall beg leave to introduce a few preliminary remarks.

I believe it is very generally admitted, that, in the canine race at least, when a bite has been inflicted by a mad dog upon another, the saliva inserted at the same moment has the power, after lying dormant a certain time, of entering into the habit, and of vitiating the saliva, of the bitten dog, and of rendering it, in its turn, equally virulent and infectious. Nor does there appear any reason to believe, at least it has never been either asserted, or attempted to be proved, that in any of the fluids secreted in the rabid animal, except the saliva, a similar noxious principle exists; in fact, its virulent effects seem wholly confined to that secretion.

Now, if we compare the saliva of a man and dog in health, they have really a great similitude in their nature; and, supposing them subject to the same laws, there may surely be a considerable degree of probability in supposing, that, when a rabid animal bites one of the human race, and he in process of time becomes rabid, the same vitiated state of of the saliva exists in him; that the virus is wholly confined to the secreting surfaces and glands of the throat; and that, like the dog, all his other secretions are equally as innoxious as in health.

But, in order to determine whether, in these conclusions, analogy may have deceived us, I earnestly recommend that the earliest opportunity be embraced of putting it to the test of experiment, by inoculating a dog with the saliva of a human being labouring under the disorder. Should rabies succeed, the question will be set at rest. Saliva when most redundant, near the fatal issue, to be selected for trial.

I, however, am anxious to proceed still further. As vitiated secretions have been accurately examined,—the urine to wit,—and a better mode of treating calculous complaints has been suggested by the research, I must strongly enforce another experiment to be made respecting this virus, namely, carefully to examine, by chemical tests, the saliva of a rabid animal, as also that of a human being labouring under the same complaint; to mark in what circumstances they may agree or differ from each other, and also to point out what constitutes the difference between the two and healthy saliva. From such an examination, should we be enabled to detect the principle which constitutes the virus, and to point out how far it may depend upon an acrid volatile salt, or an acid. We might then be able, by proper remedies, to neutralize it, and thus render it inert. That this virus is of an acid nature, is earnestly contended for by Dr. Heysham, in his Inaugural Thesis; 1777; Edinb.

Till these two (I must deem them important) experiments shall have been made, we must be content to be led, in some degree at least, by analogy. Taking it, therefore, for granted that the saliva in the human being, when rabid, is equally infectious with that of the dog, I shall go a step further; and, supposing the virus to be an acid, I shall place my chief reliance on the use of an alkali, for the purpose of neutralizing it. I confess I place more confidence in it, from a remark in the 3d vol. of Dr. Ferriar's Medical Histories, where it is asserted by Mr. Simmons that forty persons, bitten by mad dogs, applied, within a fortnight, at the Manchester Infirmary; that, being treated by the Kali purum applied to the wounded parts, they all escaped the disease; and that the same success has attended the application of this remedy in his practice during twenty years. He recommends its use to the parts which have been even treated with excision. As, from this account, he appears to have depended upon its efficacy *alone*, without excision, it certainly

2 affords affords some grounds to believe that it acts powerfully on the virus.

I shall now proceed briefly to state, under the five following heads, the particular objects I have in view in the treatment I should adopt, in a case of this dreadful malady, and also other means and remedies that appear best adapted, at least in my estimation, to fulfil the proposed intentions.

1st. To discharge, as much as possible, the saliva which probably contains the virus.

2dly. To endeavour to neutralize it, and thus to render it innoxious.

3dly. To support the patient by every means in our power.

4thly. To keep him in as perfectly a tranquil state as his circumstances and situation will admit, during the eventful struggle.

5thly. That, as not more than three or four days are allowed for the trial of our remedies, to proportion them to the impending danger.

To answer the first indication, as soon as the fact of hydrophobia is satisfactorily established, I should commence with an emetic, two or three grains of emetic tartar in a pill, for the sole purpose of evacuating the vitiated saliva from the stomach, throat, and fauces; and this I should repeat night and morning. The good effects which appear to have been derived in Dr. Satterley's case from an emetic were, probably, owing to its having answered the above purpose.

To fulfil the second object, I should recommend salt of tartar and sugar, equal parts; a portion of this to be licked up by the patient every half hour, day and night; to suffer it gradually to dissolve and mix with the saliva, which may be then expectorated.

With respect to the third indication, I should strictly forbid all sustenance by the mouth, and supply its place by a glyster to be injected every six or eight hours, composed of four ounces of Port wine, with an equal quantity of strong broth; to which may be added, as circumstances require, one drachm of laudanum. As, in cases of obstructed deglutition, the strength of the patient is thus supported for an almost indefinite period, no danger, in the short space of three or four days, can possibly arise, in the case under consideration, from inanition.

As to the fourth object, I should deem it a duty of the first consideration to avoid, as much as possible, every thing that may have the most distant tendency to produce spasm, or to agitate my patient's spirits. And here I cannot sufficiently ciently deprecate the practice that has been much too generally adopted, of constantly and repeatedly urging the unhappy sufferer to drink, to dip his hands in water, &c. &c. Every such request is, in truth, little less than so many appeals made to him to strangle himself. On the contrary, the inability to swallow having been once satisfactorily ascertained, no reason under heaven should induce us to make a second trial, until the patient demands it.

Lastly, as our time is so very short, our sheet anchor, the alkali, should be given every half hour through the day, and at least every half hour through the night, that the saliva which is constantly collecting, and greatly increases as the disorder advances, may be neutralized. The necessity of this diligent attention must be obvious, for, if I am correct in my conjecture, the saliva, as I have before hinted, both in the canine and human race, when labouring under Rabies contagiosa, will be found equally vitiated and infectious; and the salivary glands, and secreting surfaces of the throat, appear to be the only laboratory in which this virus is prepared and dispersed. This exhibits, in a strong point of view, the reason why the means hitherto adopted for the cure have uniformly failed. It is likewise greatly in favour of its locality, if I may so express myself, that the result of many careful dissections has proved most satisfactorily, that in many cases, may we not say in all? no local inflammation, nor any other assignable cause, could be detected, sufficient to account for the symptoms of this dreadful malady. The disease, it is evident, from all its symptoms and sensations, has a much nearer affinity to spasm than to inflammation; and the patient appears to fall a victim to excessive irritability. I shall only further observe, that, should the above plan not succeed, "the absolute failure," as Dr. Parry very judiciously remarks with respect to bleeding in this disease, "leaves us, as to the event, precisely where we were; and, if we can merely alleviate certain symptoms, and smooth the avenues of death, some good is doubtless obtained."

*Chichester;* WILLIAM GUY.

*March* 2, 1815.

